# Melatonin protects rats from radiotherapy-induced small intestine toxicity

**DOI:** 10.1371/journal.pone.0174474

**Published:** 2017-04-12

**Authors:** Beatriz Fernández-Gil, Ahmed E. Abdel Moneim, Francisco Ortiz, Ying-Qiang Shen, Viviana Soto-Mercado, Miguel Mendivil-Perez, Ana Guerra-Librero, Darío Acuña-Castroviejo, María M. Molina-Navarro, José M. García-Verdugo, Ramy K. A. Sayed, Javier Florido, Juan D. Luna, Luis Carlos López, Germaine Escames

**Affiliations:** 1 Instituto de Biotecnología, Centro de Investigación Biomédica, Universidad de Granada, Granada, Spain; 2 Department of Zoology and Entomology, Faculty of Science, Helwan University, Cairo, Egypt; 3 Neuroscience Research Group, Medical Research Institute, Faculty of Medicine, University of Antioquia, Medellin, Colombia; 4 Departamento de Fisiología, Facultad de Medicina, Universidad de Granada, Granada, Spain; 5 Instituto Cavanilles, Universidad de Valencia, Valencia, Spain; 6 Department of Anatomy and Embryology, Faculty of Veterinary Medicine, Sohag University, Sohag, Egypt; 7 Departamento de Bioestadística, Facultad de Medicina, Universidad de Granada, Granada, Spain; Northwestern University Feinberg School of Medicine, UNITED STATES

## Abstract

Radiotherapy-induced gut toxicity is among the most prevalent dose-limiting toxicities following radiotherapy. Prevention of radiation enteropathy requires protection of the small intestine. However, despite the prevalence and burden of this pathology, there are currently no effective treatments for radiotherapy-induced gut toxicity, and this pathology remains unclear. The present study aimed to investigate the changes induced in the rat small intestine after external irradiation of the tongue, and to explore the potential radio-protective effects of melatonin gel. Male Wistar rats were subjected to irradiation of their tongues with an X-Ray YXLON Y.Tu 320-D03 irradiator, receiving a dose of 7.5 Gy/day for 5 days. For 21 days post-irradiation, rats were treated with 45 mg/day melatonin gel or vehicle, by local application into their mouths. Our results showed that mitochondrial oxidative stress, bioenergetic impairment, and subsequent NLRP3 inflammasome activation were involved in the development of radiotherapy-induced gut toxicity. Oral treatment with melatonin gel had a protective effect in the small intestine, which was associated with mitochondrial protection and, consequently, with a reduced inflammatory response, blunting the NF-κB/NLRP3 inflammasome signaling activation. Thus, rats treated with melatonin gel showed reduced intestinal apoptosis, relieving mucosal dysfunction and facilitating intestinal mucosa recovery. Our findings suggest that oral treatment with melatonin gel may be a potential preventive therapy for radiotherapy-induced gut toxicity in cancer patients.

## Introduction

Mucositis is a debilitating and untreatable condition involving deep mucosal ulcerations [1]. It is among the most common toxicities associated with cytotoxic cancer therapy, imposing a substantial burden on patients undergoing chemotherapy and radiation treatments. Mucositis is caused when cytotoxic cancer treatment damages normal tissue, and can broadly impact various mucosal tissues [2]. Oral mucositis is associated with a cascade of events in multiple tissue regions [3–5], with radiotherapy-induced gut toxicity being a prevalent dose-limiting toxicity that manifests following radiotherapy [6].

In the clinical setting, radiation-induced damage to healthy intestine tissue is a common side-effect caused by out-of-field or scattered radiation. Since it is a rapid turnover system, the small intestine mucosa is a focus for radiation related side-effects [7]. The severity of intestinal radiation toxicity is determined based on the extent of radiation-induced intestinal crypt cell death. Direct radiation to the small intestine induces changes in cellular function, that can lead to mucosal breakdown, microcirculation damage [8], leukocyte cell adhesion and emigration, and other effects [9,10]. Some of these pathophysiological manifestations in the small intestine have also been described as out-of-field effects of external beam irradiation of other organs [7]. Thus, the prevention of radiation enteropathy requires protection of the small intestine. However, despite the prevalence and burden of radiotherapy-induced gut toxicity, its pathophysiological pathways are yet unclear, and there is currently no effective treatment for this disease.

Melatonin (N-acetyl-5-methoxytryptamine) is a potent free radical scavenger with anti-oxidant and anti-inflammatory properties [11–15]. It reportedly maintains mitochondrial homeostasis under various experimental conditions [16–19], and protects against radiation injury [20–23]. We recently found that melatonin could completely prevent the activation of multiple radiation-induced pathways associated with oral mucositis [24]. In our previous report, we describe the involvement of mitochondrial oxidative stress, bioenergetic impairment, and subsequent NLRP3 (NLR-related protein 3 nucleotide-binding domain leucine-rich repeat containing receptors-related protein 3) inflammasome activation in oral mucositis development following tongue irradiation. Moreover, the application of a melatonin gel prevented mucosal disruption and ulcer formation, protecting the mitochondria from radiation damage and blunting NF-κB/NLRP3 inflammasome signaling activation in the tongue. Phase II clinical trials are now investigating melatonin gel in patients with head and neck cancer who are undergoing chemoradiation, involving cisplatin, carboplatin, or cetuximab plus radiation given in fractionated doses of 2 Gy per day Monday through Friday (10 Gy per week for a total dose of 60–70 Gy).

The presently available data suggest that melatonin is protective against oxidative stress and organ injury, including radiotherapy-induced mucositis. However, the development of effective treatment is hindered by our lack of knowledge regarding the mechanisms involved in radiotherapy-induced gut toxicity. Thus, our present study aimed to investigate the changes induced in rat small intestine following external irradiation of the tongue, and to explore the potential radio-protective effects of a melatonin gel.

## Materials and methods

### Animal irradiation

**Fig 1:**
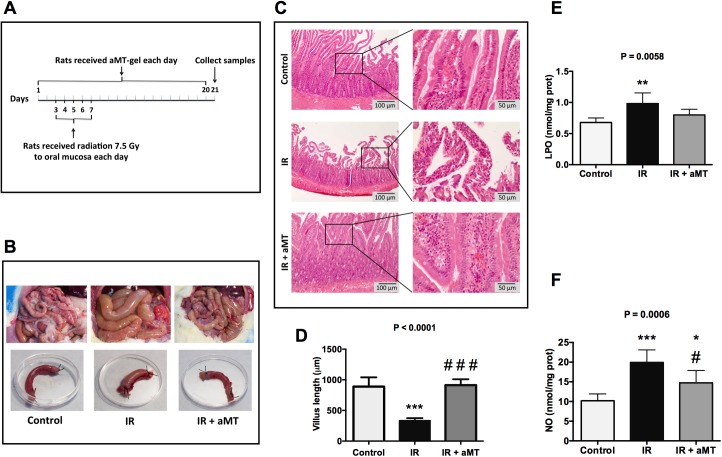
Effects of melatonin gel on small intestine damage induced by external radiation in rats. Schedule of fractionated radiation and melatonin treatment (A). Representative pictures of small intestine from non-irradiated rats (control) and irradiated rats treated with vehicle (IR) or melatonin gel (IR + aMT) (B). H&E staining of the small intestine (C) and villus length (D) from non-irradiated rats (control) and irradiated rats treated with vehicle (IR) or melatonin gel (IR + aMT). The right panels show details at higher magnification. Scale bar = 100 μm and 50 μm in the left and right panels, respectively. LPO (E) and NO levels (F) in small intestine tissue homogenate from non-irradiated rats (control) and irradiated rats treated with vehicle (IR) or melatonin gel (IR + aMT); *n* = 4 per group. Data are expressed as mean ± s.e.m. *P < 0.05, **P < 0.01, ***P < 0.001 vs. control, and ^#^*P* < 0.05 vs. IR.

All experiments were performed according to a protocol approved by the Institutional Animal Care and Use Committee of the University of Granada (procedures CEEA-2009-238) and were in accordance with the European Convention for the Protection of Vertebrate Animals used for Experimental and Other Scientific Purposes (CETS # 123) and the Spanish law (R.D. 53/2013). This study was conducted using adult (3-month-old) male Wistar rats. The rats used in this study are the same as in reference 24 [24]. They were maintained in the University’s facility with a 12-hour light/12-hour dark cycle at 22 ± 2°C, with lights on at 08:00 h (ZT = 0), and they received water and food *ad libitum*. The rats were randomly divided into three groups: control (non-irradiated), irradiated + vehicle (melatonin-free gel), and irradiated + local application of 3% melatonin gel. For this study, we used a gel containing 3% melatonin (Fagron Ibérica SAU, Terrasa, Spain) dissolved in 0.3% ethanol:Pluronic F-127 (Fagron Ibérica SAU, Terrasa, Spain). The gel was made in our laboratory and it is under patent. Previously, we performed a dose-response study and the maximal therapeutic effect was obtained with 3% melatonin gel [24]. Therefore, in the group that received melatonin gel, the total melatonin dose was 45 mg/day for 21 days. A group of irradiated rats was also treated with vehicle, consisting in a 0.3% ethanol:Pluronic F-127 melatonin-free gel. Rats were anesthetized with 1 mL equithesin/kg body weight, intraperitoneal (i.p.), prior to irradiation with a dose of 7.5 Gy/day for five consecutive days (Fig 1A) [24]. Irradiation was always applied in the morning between ZT 1 and ZT 4. Ionizing radiation was delivered using an X-Ray YXLON Y. Tu 320-D03 irradiator (Yxlon, Hamburg, Germany) with a voltage of 207.3 kV, working current of 10.5 mA, 5-mm-diameter irradiation focus, 0.25-mm Cu filter system, 15-cm target distance, irradiation field of 0.78 cm^2^, and delivered dosage of 100.2 cGY/min. For irradiation, each animal was placed in the irradiator with a standardized snout positioning, and with a lead shield covering the entire animal except for the mouth. The radiation dose to shielded areas was 2.4 × 10^−2^ cGy/min (7.5 min per rat), ensuring that the total body dose received by rats during irradiation treatment was less than 9.0 × 10^−3^ Gy. After radiation, animals that received the same treatment were housed with four or five animals per cage.

Melatonin gel or vehicle was applied in the oral cavity starting 48 hours before the first irradiation dose, until up to 14 days after the last irradiation exposure. Using a plastic Pasteur pipette, the gel was applied in the intraoral regions three times per day (ZT 0, ZT 6, and ZT 12). On days of irradiation, the gel was applied 1 hour before and 1 hour after each radiation dose, with the third dose 6 hours later. Rats were monitored daily, and euthanized under an over dose of anesthesia (>1.5 mL equithesin/kg body weight, i.p.) followed by cervical dislocation, 14 days after the last radiation dose. All the animals were sacrificed in the morning between ZT 0 and ZT 4. Upon sacrifice, the animals’ small intestines were harvested and processed as described below for each analysis/technique.

### Histology, histopathology, and electron microscopy

Small intestine samples were fixed in 10% buffered formalin for 48 hours, embedded in paraffin, and sliced into 4-μm-thick sections. Sections were deparaffinized with xylene and stained with hematoxylin and eosin (H&E).

In these same sections, we then performed immunohistochemistry using primary rabbit polyclonal antibody to zonula occludens-1 (ZO-1) (MABT11, 1:50; Millipore, Madrid, Spain) and primary rabbit antibody Ki-67 (Ab16667, 1:100; Abcam, Cambridge, United Kingdom). For rabbit primary antibodies, we used the Vectastain ABC kit (Vector PK6101; Burlington, NC, USA). For qualitative identification of antigens by light microscopy, we used the peroxidase substrate DAB kit (Vector SK-4100; Thermo Scientific, Madrid, Spain). Sections were examined using an OLYMPUS CX41 microscope, and images were scanned under equal light conditions using the CELL A program (Olympus,Hicksville,NY,USA). The epithelia region was selected based on its histological appearance, and the same region was used for all immunohistochemistry studies. Ki-67 activity was quantified using ImageJ 1.46r, with the Cell Counter plugin. The labeling percentage was assessed as the ratio of the number of cells stained with Ki-67 to the total number of cells counted per section. ZO-1 expression was also quantified using ImageJ 1.46r software. Briefly, immunohistochemistry images were acquired using identical parameters, the figures were transformed into 8-bit images, and background was subtracted, and the ZO-1 staining intensity was measured. The reported values are the average of different images from four different rats (at least ten images from each rat).

For electron microscopy, rats were perfused with physiologic saline (0.9% NaCl) and fixation solution (2% paraformaldehyde and 2% glutaraldehyde). Small intestine samples were fixed with 2% osmium, rinsed, dehydrated, and embedded in Durcupan resin (Fluka; Sigma-Aldrich, St. Louis, USA). Ultra-thin sections (0.08 μm) were cut using a diamond knife, and then stained with lead citrate (Reynolds solution). Samples were analyzed using a Leo 906E transmission electron microscope (Carl Zeiss, Jena, Germany) equipped with a Morada digital camera (Silicon Integrated Systems, Hsinchu, Taiwan).

### Isolation of pure mitochondria, nuclei, and cytosol

After the animals were sacrificed, we immediately isolated the small intestine mitochondria, nuclei, and cytosol by differential centrifugation and Percoll density gradient as described previously [25].

### Measurement of LPO and NOx levels

Tissues were weighed and then homogenized in ice-cold 50 mM Tris-HCl buffer (pH 7.4). The homogenate was centrifuged at 800 × *g* for 10 min at 4°C. Some aliquots of the supernatant were stored at −80°C for total protein determination [26], while other aliquots were used for lipid peroxidation (LPO) or nitrite plus nitrate (NOx) measurement, a marker for NO levels. LPO was assessed using the Bioxytech LPO-568 assay kit (OxisResearch, Portland, OR, USA) [27]. LPO levels were expressed as nmol/mg protein. NOx levels were determinate using the Griess reaction [28], and NOx concentrations were expressed as nmol nitrite/mg protein.

### Measurement of GPx, GRd, and Mn-SOD2 activities

To measure glutathione peroxidase (GPx) activity, small intestine mitochondria were incubated for 4 min at 37°C in a working solution, containing buffer A plus 4 mM sodium azide, 4 mM GSH, 0.2 mM NADPH, and 0.5 U/mL GRd. After incubation, the reaction was started with the addition of cumene hydroperoxide (0.3%). GPx activity was determined by following NADPH oxidation at 340 nm for 3 min in a UV spectrophotometer (Shimadzu Deutschland GmBH, Duisburg, Germany) [29]. To measure glutathione reductase (GRd) activity, samples were incubated for 4 min at 37°C in a working solution containing buffer A plus 2 mM GSSG. Then, the reaction was started by adding 9.5 mM NADPH solution. GRd activity was measured by following NADPH oxidation at 340 nm for 3 min. For both measurements, nonenzymatic NADPH oxidation was subtracted from the overall rates. GPx and GRd activities were expressed as nmol/min/mg protein. Mn superoxide dismutase (SOD2) was assayed in terms of its ability to inhibit the auto-oxidation of adrenaline to adrenochrome at pH 10.2 [30]. SOD2 activity was expressed as U/mg protein, where 1 unit equals 50% inhibition of epinephrine auto-oxidation.

### Measurement of GSH and GSSG levels

Glutathione (GSH) and glutathione disulfide (GSSG) were measured using an established fluorometric method [31]. Small intestine mitochondria were sonicated in ice-cold 50 mM Tris-HCl buffer (pH 7.4), and then centrifuged at 800 × *g* for 10 min at 4°C. Aliquots of the supernatant were deproteinized using ice-cold 10% trichloroacetic acid, and then centrifuged at 20,000 × g for 15 min. For GSH measurement, supernatant aliquots were incubated for 15 minutes at room temperature with an ortho-phthalaldehyde/ethanol solution (1 mg/mL) and phosphate buffer (100 mM sodium phosphate and 5 mM EDTA-Na2, pH 8.0). Then the sample fluorescence was measured at 340 nm excitation and 420 nm emission wavelengths using a plate-reader spectrofluorometer (Bio-Tek Instruments, Inc., Winooski, VT, USA).

For GSSG measurement, supernatant aliquots were preincubated with N-ethylmaleimide solution (5 mg/mL) at room temperature for 40 min, and then alkalinized with 0.1 N NaOH. Aliquots of this mixture were then incubated for 15 minutes at room temperature with ortho-phthalaldehyde solution (1 mg/mL) and 0.1 N NaOH, and the sample fluorescence was then measured. GSH and GSSG concentrations were expressed as nmol/mg protein.

### Western blot analysis

We performed protein extraction and western blot analyses as described previously [32]. The utilized antibodies included rabbit antibody to GPx (sc-30147, 1:500; Santa Cruz Biotechnology, Santa Cruz, CA, USA), GRd (sc-32886, 1:200; Santa Cruz Biotechnology), Mn-SOD (sc-30080, 1:500; Santa Cruz Biotechnology), p53 (sc-6243, 1:200; Santa Cruz Biotechnology), Bax (sc-526, 1:200; Santa Cruz Biotechnology), Bcl2 (sc-492, 1:200; Santa Cruz Biotechnology), ASC (AG-25B-0006-C100, 1:1,000; AdipoGen, San Diego, CA, USA), caspase 1 (sc-22165, 1:500; Santa Cruz Biotechnology), IL-1β (sc-7884, 1:100; Santa Cruz Biotechnology), COX-2 (sc-7951, 1:200; Santa Cruz Biotechnology); mouse antibody to OXPHOS (MitoProfile Total OXPHOS Rodent WB Antibody Cocktail, MS604, 1:250; Mitosciences, Eugene, OR, USA), NLRP3 (AG-20B-0014, 1:500; AdipoGen), NFκB (p65) (sc-8008, 1:100; Santa Cruz Biotechnology), TNFα (sc-52746, 1:200; Santa Cruz Biotechnology) and GAPDH (sc-25778, 1/500; Santa Cruz Biotechnology); goat antibody to pro-caspase 1 (sc-622, 1:500; Santa Cruz Biotechnology); goat anti-rabbit IgG (Pierce® Antibody 31460, 1:5,000; Thermo Scientific); HRP goat anti-mouse IgG (554002, 1:1,000; BD PharmigenTM, San Jose, CA, USA); and donkey anti-goat IgG-HRP (sc-2020, 1:1,000; Santa Cruz Biotechnology). The proteins were visualized using a Western Lightning Plus-ECL chemiluminescence kit (PerkinElmer, Billerica, MA, USA) following the manufacturer’s protocol. Images were analyzed using the Kodak Image Station 2000R (Eastman Kodak Company, Rochester, NY, USA). Protein bands intensity were normalized to GAPDH, and the data expressed in terms of percent relative to controls.

### Melatonin determination by HPLC

Melatonin was extracted using trichloromethane, and the organic phase was evaporated to dryness using a SPD 2010 Speed Vac System (Fisher Scientific, Madrid, Spain). Samples were analyzed by HPLC (Shimadzu Europe GmbH, Duisburg, Germany) with a Waters Sunfire C18 column (150 × 4.5 mm, 5 μm). Melatonin fluorescence was measured in a Shimadzu RF-10A XL fluorescence detector (Shimadzu Europe GmbH, Duisburg, Germany), with excitation and emission wavelengths of 285 and 345 nm, respectively [32].

### Statistical analysis

A one-way Analysis of Variance (ANOVA) was performed to assess differences among means and when significant pairwise comparisons were carried out using Tukey´s penalizations. All tests were declared significant at 0.05 error but *P* values were reported in all the cases. STATA 14.1 was used for statistical computations. Data are presented as mean ± s.e.m.

## Results

### Melatonin gel prevents deleterious effects of external radiation in small intestine epithelia

Although the intestine was outside of the irradiation field, macroscopic changes in the small intestine were apparent at 21 days after tongue irradiation with 5 Gy/day for 5 consecutive days (Fig 1A and 1B; Table A in S1 File). Typical signs of mucositis were detectable in the small intestines of irradiated animals, but not in the rats treated with melatonin gel. H&E staining was performed to assess the tissue damage after irradiation (Fig 1C). IR-treated animals exhibited significant loss of crypt architecture, severe villous epithelial atrophy, villus degeneration and shortening, and marked infiltration of the lamina propia with inflammatory cells (Fig 1C and 1D). Animals treated with melatonin gel showed reduced intestinal morphological changes, including increased villi and reductions of cellular infiltration and areas of hemorrhage (Fig 1C and 1D; Table A in S1 File).

**Fig 2:**
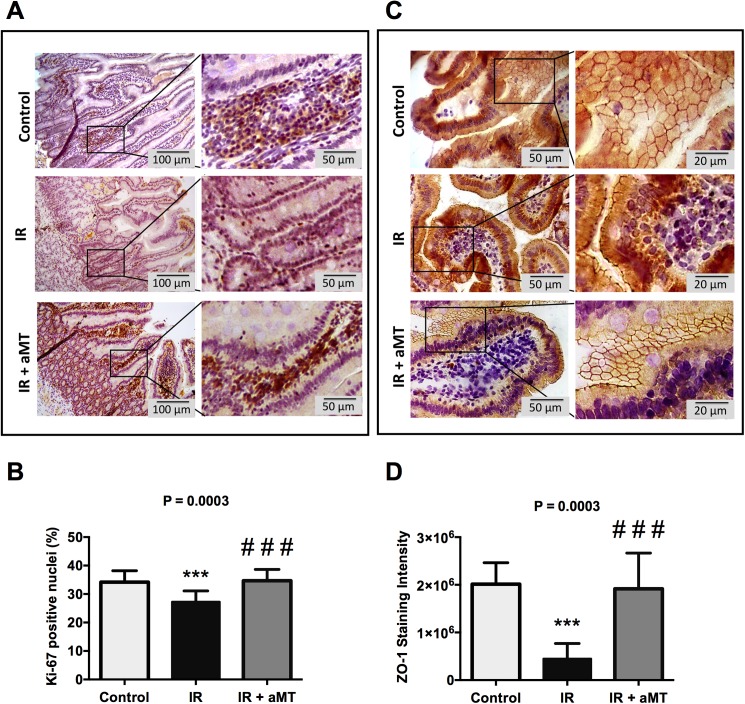
Immunohistochemical staining of Ki-67 and ZO-1. Immunohistochemical staining of the proliferation marker Ki-67 (A) and of the tight junctions protein zonula occludens 1 (ZO-1) (C) in rat small intestine from non-irradiated rats (control) and irradiated rats treated with vehicle (IR) or melatonin gel (IR + aMT). The right panels show details at higher magnification. Scale bar = 100 μm and 50 μm in the left and right panels, respectively. (B, D) Quantification of the staining in A and C; n = 4 per group. Data are expressed as mean ± s.e.m. ***P < 0.001 vs. control and ###*P* < 0.001 vs. IR.

Oral irradiation to rats also resulted in increased small intestinal damage associated with oxidative stress, as indicated by enhanced LPO (*P* < 0.01) and NO (*P* < 0.001) compared to non-irradiated controls. Treatment with melatonin gel decreased oxidative stress, especially NO levels, (*P* < 0.05) (Fig 1E and 1F; Table A in S1 File). Since the intestinal epithelium is one of the fastest proliferating tissues in the body [33], it is particularly vulnerable to reactive oxygen species (ROS) and, therefore, to radiotherapy. Based on the evidence of increased oxidative stress in the small intestine (Fig 1E and 1F), proliferating cells were identified by immunohistochemical staining of Ki-67 (Fig 2A and 2B). Irradiated rats showed decreased numbers of Ki-67-positive cells (*P* < 0.01), and the group treated with melatonin gel showed greater numbers of Ki-67-positive cells compared to the irradiated group (*P* < 0.01) (Fig 2A and 2B; Table B in S1 File).

To confirm the association between ROS, loss of proliferation, and intestinal barrier dysfunction, we performed an immunohistochemical analysis of the tight junction (TJ) protein ZO-1 in rat small intestine tissue (Fig 2C and 2D; Table B in S1 File). We found that tongue irradiation significantly decreased ZO-1 levels (*P* < 0.01) in rat small intestine compared to in the non-irradiated group. Moreover, treatment with melatonin gel completely counteracted this effect (*P* < 0.01).

### Melatonin gel prevents external radiation-induced mitochondrial dysfunction in small intestine epithelia

**Fig 3:**
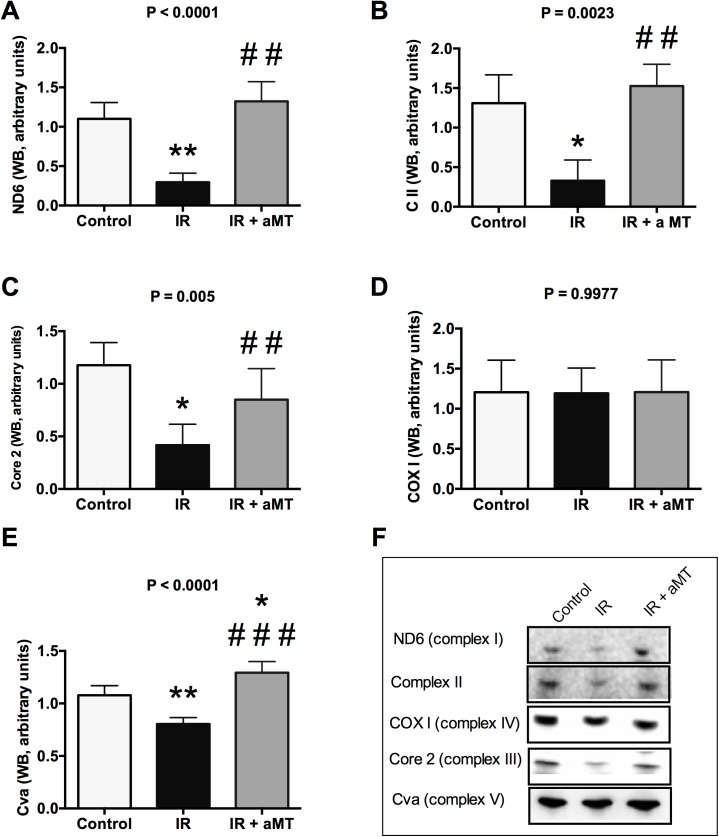
OXPHOS analysis. Western blot (WB) analysis and densitometric quantification of OXPHOS: ND6 (complex I) (A), complex II (B), core II (complex III) (C), COX I (complex IV) (D), Cva (complex V) and Western blot (WB) analysis of OXPHOS (F) in the mitochondria of small intestine cells from non-irradiated rats (control) and irradiated rats treated with vehicle (IR) or melatonin gel (IR + aMT); n = 6 per group. Data are expressed as mean ± s.e.m. *P < 0.05, **P < 0.01 vs. control; and ##*P* < 0.01, ###*P* < 0.001 vs. IR.

Interestingly, the oxidative stress in the small intestine caused by tongue irradiation was associated with increased mitochondrial damage. Our analysis of bioenergetic capacity revealed that compared to non-irradiated rats, the irradiated rats showed significantly reduced expressions of respiratory complexes I, II, and III, as well as the ATP synthase (*P* < 0.001, *P* < 0.5, *P* < 0.5, and *P* < 0.01, respectively) (Fig 3A–3F; Table C in S1 File). Treatment with melatonin gel completely counteracted this mitochondrial dysfunction, restoring the levels of the complexes I, II, III, and V to above the levels in controls (*P* < 0.01, *P* < 0.01, *P* < 0.01, and *P* < 0.001, respectively) (Fig 3A–3F).

**Fig 4:**
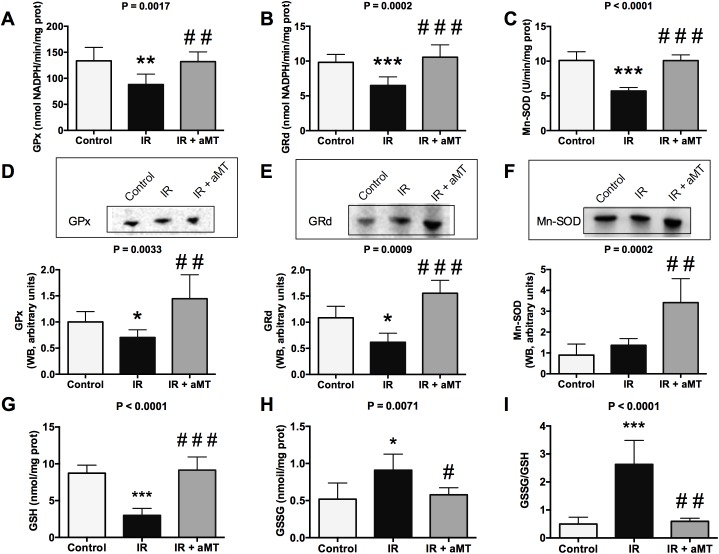
Oxidative stress analysis. Activity and western blot (WB) analysis of GPx (A and D), GRd (B and E), and Mn-SOD (C and F) in the mitochondria of small intestine cells from non-irradiated rats (control) and irradiated rats treated with vehicle (IR) or melatonin gel (IR + aMT). GSH (G) and GSSG levels (H), and GSSG/GSH ratio (I) in the mitochondria of small intestine cells. n = 6 per group. Data are expressed as mean ± s.e.m. *P < 0.05, **P < 0.01, ***P < 0.001 vs. control; and ^#^*P* < 0.05, ^##^*P* < 0.01, ^###^*P* < 0.001 vs. IR.

Mitochondrial dysfunction leads to enhanced production of ROS and reactive nitrogen species (as shown in Fig 1E and 1F), inducing cell death. We observed that radiation decreased the activity and protein levels of mitochondrial GPx (Fig 4A and 4D) (*P* < 0.01 and *P* < 0.05) and GRd (Fig 4B and 4E; Table D in S1 File) (*P* < 0.001 and *P* < 0.05). Irradiation also decreased the SOD2 activity (Fig 4C) (*P* < 0.001) without altering the protein levels (Fig 4F). Melatonin gel treatment significantly increased the activity and protein levels of GPx (Fig 4A and 4D) (*P* < 0.01 and *P* < 0.01), GRd (Fig 4B and 4E; Table D in S1 File) (*P* < 0.001 and *P* < 0.001), and SOD2 (Fig 4C and 4F) (*P* < 0.001 and *P* < 0.01).

**Fig 5:**
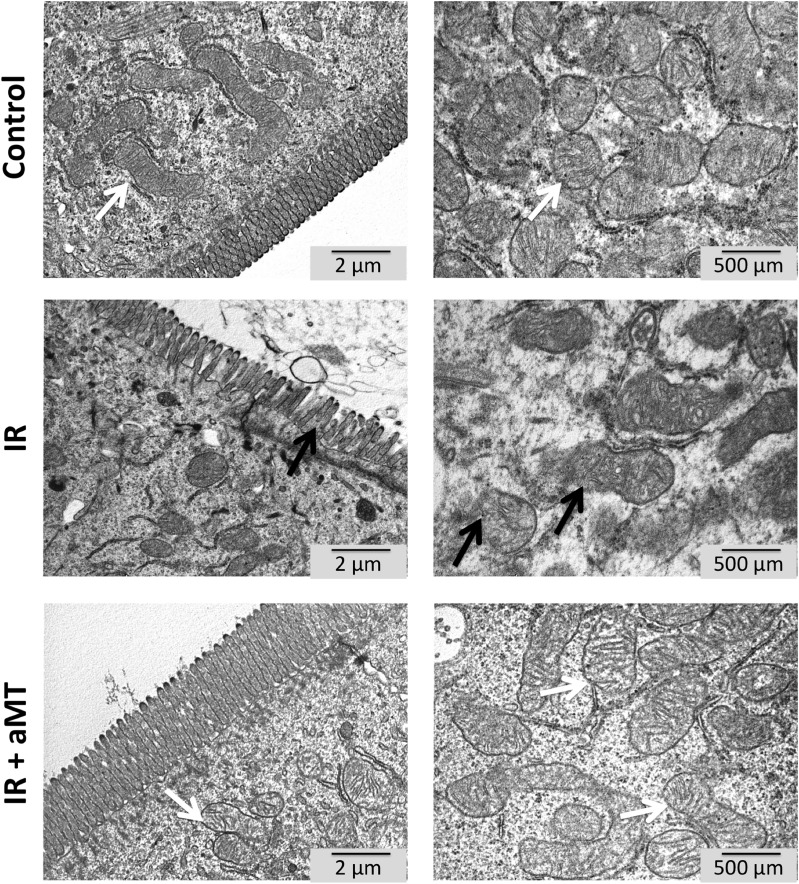
Electron microscopy (EM) analysis. EM images of mitochondria of small intestine cells from non-irradiated rats (control) and irradiated rats treated with vehicle (IR) or melatonin gel (IR + aMT); n = 4 per group. Swollen and less dense mitochondria are indicated by black arrows. Normal mitochondria are indicated by white arrows. Degenerated and shorted villi are indicated by black arrows. Right panels show details at higher magnification. Scale bar = 2 μm and 500 nm in the left and right panels, respectively.

Our results suggested that small intestine mitochondria showed an oxidizing response to oral radiation, which was further supported by the increased mitochondrial GSSG/GSH ratio (*P* < 0.001) (Fig 4I). Treatment with melatonin gel decreased the oxidative stress in the small intestine characterized by increased GSH levels (Fig 4G) (*P* < 0.001), restoring a normal GSSG/GSH ratio (*P* < 0.001) (Fig 4I). These findings were in line with our observation that melatonin treatment reduced mitochondrial dysfunction in the small intestine and could thus reduce mitochondrial damage. To confirm this hypothesis, we analyzed mitochondrial structure using EM (Fig 5). Compared to non-irradiated rats, irradiated rats showed cells with abundant broken and swollen mitochondria, poor cristae, and numerous vacuoles. Melatonin gel administration completely prevented this mitochondrial disintegration induced by external radiation. This suggests that mitochondrial damage could be involved in radiotherapy-induced gut toxicity [24]. We also observed that shortened and degenerated villi completely recovered with melatonin gel treatment.

### Melatonin gel prevents external radiation-induced NLRP3 inflammasome pathway activation in small intestine epithelia

**Fig 6:**
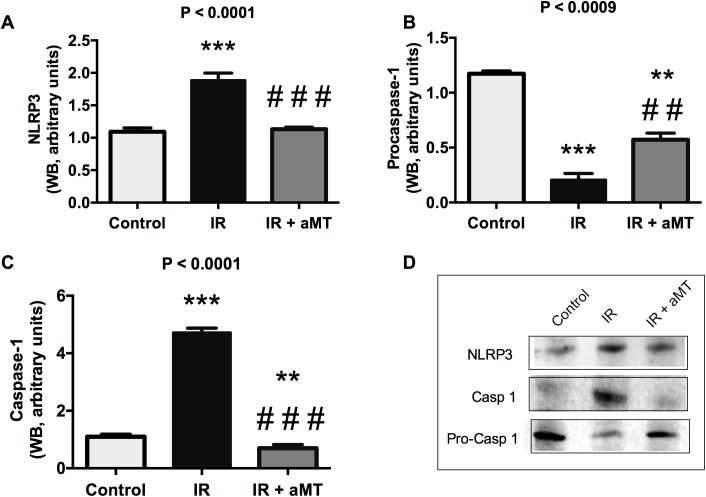
NLRP3 inflammasome pathway. Western blot (WB) analysis and densitometric quantification of NLRP3 (A, D), pro-caspase 1 (B, D), and caspase 1 (C, D) in small intestine from non-irradiated (control) and irradiated rats treated with vehicle (IR) or melatonin gel (IR + aMT); n = 6 per group. Data are expressed as mean ± s.e.m. **P < 0.01, ***P < 0.001 vs. control; and ##*P* < 0.01, ###*P* < 0.001 vs. IR.

**Fig 7:**
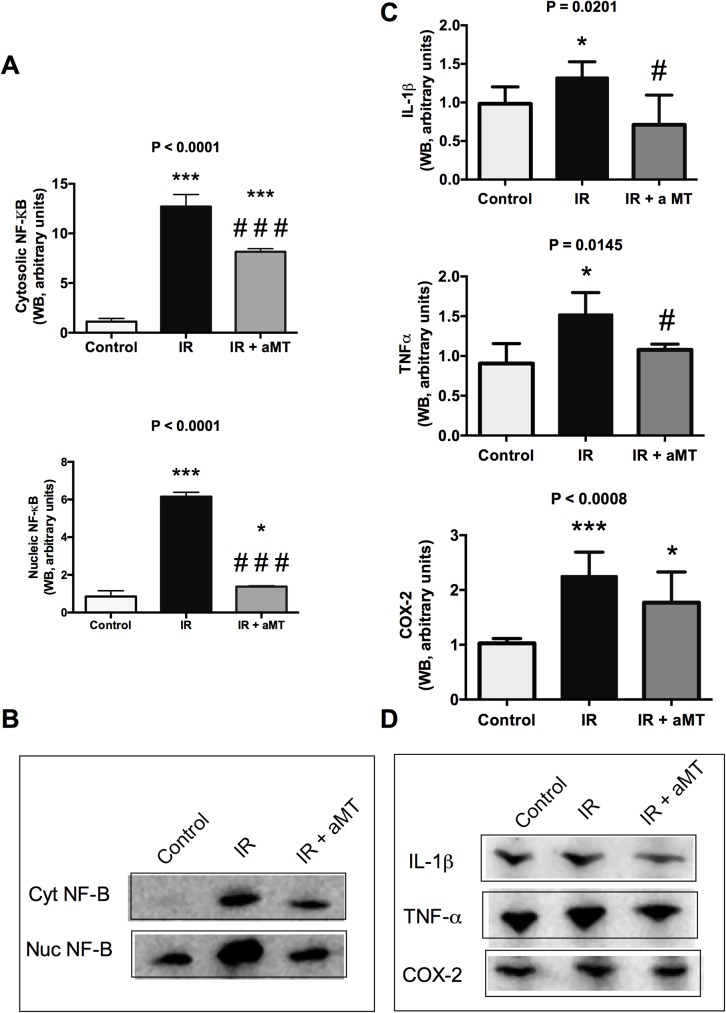
NF-κB pathway. Western blot (WB) analysis and densitometric quantification of NF-κB subunit p65 in nuclear and cytosolic fractions (A, B); cellular inflammatory cytokines IL-1β, TNF-α and the enzyme COX-2 (C, D) from small intestine cells from non-irradiated (control) and irradiated rats treated with vehicle (IR) or melatonin gel (IR + aMT); n = 6 per group. Data are expressed as mean ± s.e.m. *P < 0.05, ***P < 0.001 vs. control; and #*P* < 0.05, ###*P* < 0.001 vs. IR.

It was recently reported that mitochondrial ROS may promote inflammation by activating the multiprotein cytoplasmic complexes inflammasome NLRP3 [34,35]. NLRP3 inflammasome activation leads to caspase-1 activation and subsequent cleavage of pro-cytokines, such as IL-1β, into their mature forms. This inflammasome is also reportedly involved in irradiation-induced mucositis [24]. In agreement with these prior studies, our present results revealed significantly increased NLRP3 in the small intestine (*P* < 0.001) following tongue irradiation compared to in non-irradiated controls (Fig 6A and 6D). Moreover, irradiation led to decreased protein levels of pro-caspase-1 (Fig 6B and 6D) (*P* < 0.001), reflecting its activation to caspase-1 (Fig 6C and 6D; Table E in S1 File) (*P* < 0.001) by activated NLRP3, and yielding a significant increase in IL-1β (*P* < 0.05) (Fig 7C and 7D; Table F in S1 File). Treatment with melatonin gel completely suppressed inflammasome activation in the small intestine (Fig 6A–6D), decreasing IL-1β levels (*P* < 0.05) (Fig 7C and 7D).

### Melatonin gel prevents external radiation-induced NF-kB pathway activation in small intestine epithelia

NLRP3-activated caspase-1 requires the substrate pro-IL-1ß, which is produced by the NF-κB pathway. Thus, we evaluated the contribution of the nuclear factor kappa B (NF-κB) pathway to irradiation-induced small intestine toxicity (Fig 7A and 7B; Table F in S1 File). Irradiation significantly increased the protein levels of NF-kB in the cytosol and nuclei (*P* < 0.001 in both) in the small intestine compared to in the non-irradiated group, and treatment with melatonin gel suppressed the irradiation-activated NF-κB pathway. NF-kB activation leads to the expression of up to 200 inflammatory response-related genes. Our present results confirmed that irradiation led to increased protein levels of IL-1β, TNFα, and COX-2 (*P* < 0.05, *P* < 0.05, and *P* < 0.001, respectively) in the small intestine (Fig 7C and 7D). We further observed significant reductions of these inflammatory mediators of the NF-κB response following treatment with melatonin gel (Fig 7C and 7D; Table F in S1 File).

### Melatonin gel prevents external radiation-induced apoptosis in small intestine epithelia

**Fig 8:**
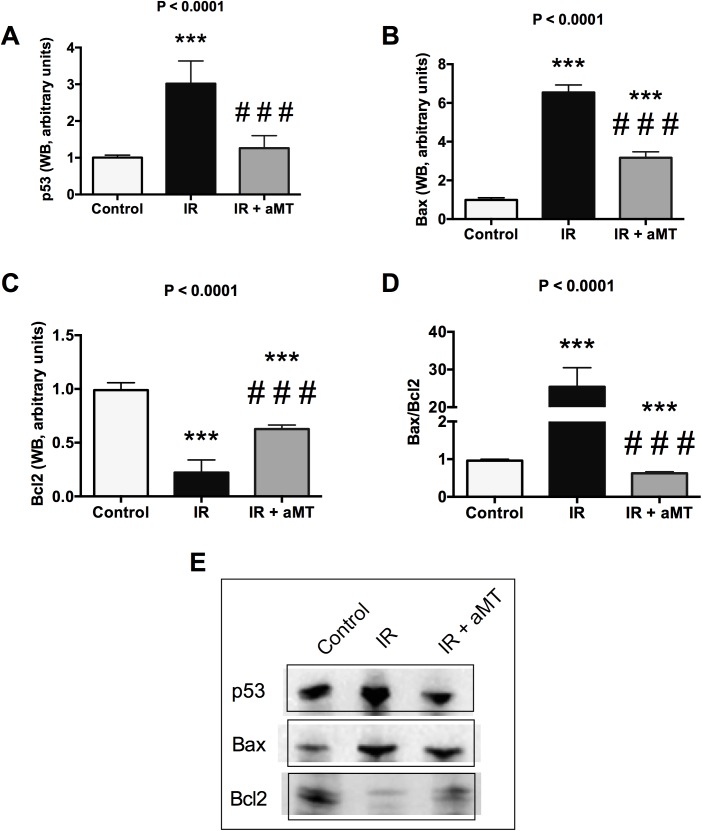
Apoptotic pathway. WB analysis and densitometric quantification of p53 (A), Bax (B), and Bcl2 (C), along with the Bax/Bcl2 ratio (D) in small intestine from non-irradiated rats (control) and irradiated rats treated with vehicle (IR) or melatonin gel (IR + aMT); n = 6 per group. Data are expressed as mean ± s.e.m. ***P < 0.001 vs. control and ###*P* < 0.001 vs. IR.

The inflammatory mediators produced by NF-κB pathways substantially increase apoptosis, decrease proliferation, and degrade the extracellular matrix [1,5,36]. Increased apoptosis is also seen in relation to the mitochondrial dysfunction and increased ROS caused by irradiation [24]. Compared to non-irradiated controls, irradiation effectively upregulated p53 (Fig 8A and 8E; Table G in S1 File) (*P* < 0.001), leading to a marked increase of Bax (Fig 8B and 8E) (*P* < 0.001) and decrease of Bcl2 (Fig 8C and 8E) (*P* < 0.001), reflecting significant apoptotic changes and a high degree of cellular injury. Treatment with melatonin gel significantly decreased p53 expression (Fig 8A and 8E; Table G in S1 File) (*P* < 0.001), resulting in a strong reduction of the Bax/Bcl2 ratio compared to irradiated rats (*P* < 0.001) (Fig 8D and 8E).

### Melatonin levels in small intestine epithelia

**Fig 9:**
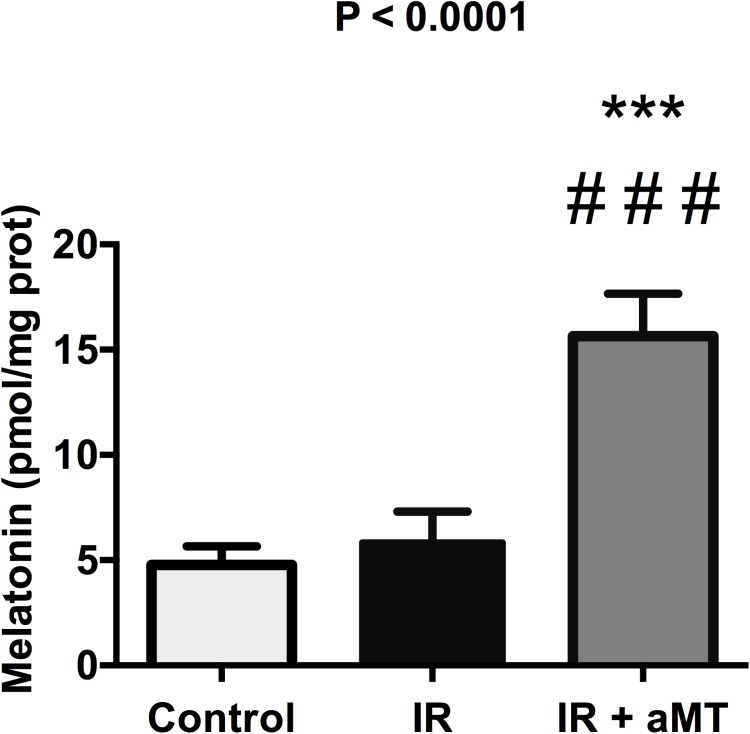
Melatonin levels. HPLC analysis of the melatonin content of the small intestine from non-irradiated rats (control) and irradiated rats treated with vehicle (IR) or melatonin gel (IR + aMT); n = 6 per group. Data are expressed as mean ± s.e.m. ***P < 0.001 vs. control and ###*P* < 0.001 vs. IR.

To investigate whether melatonin reached the small intestine to protect it against radiotherapy-induced gut toxicity *in situ*, we assessed the indoleamine content of the rats’ small intestines (Fig 9; Table H in S1 File). Compared to rats that were irradiated and not treated with melatonin, the rats treated with melatonin gel showed significantly increased melatonin content in their small intestines (*P* < 0.001), suggesting that melatonin reaches the small intestine in an adequate quantity to protect them.

## Discussion

Mucositis is an important side effect associated with radiotherapy alone or in combination with systemic therapies, and there is presently no definitive prophylaxis or treatment available [1]. Severe mucositis commonly necessitates deescalation of chemotherapy dosing or breaks in radiation treatment, both of which negatively impact a patient’s cancer treatment outcome [1].

Although it is well known that irradiation induces intestinal mucositis [37], the present report is the first to demonstrate intestinal damage induced by tongue irradiation. In our study, the small intestine was located outside of the irradiation field, but still exhibited radiation damage, including macroscopic signs of intestinal mucositis and morphological changes associated with substantial changes in intestinal architecture. Rats subjected to tongue irradiation showed signs of mucosal damage, including a significant decrease in villus height. Interestingly, the results were similar to those observed when radiation is applied directly to the intestine. Radiation affects the integrity of the clonogenic compartment, resulting in villi shortening and a loss of epithelial cell lining, severe crypt damage, cell necrosis, increased collagen deposition in the submucosa, edema of the muscle layer, and incipient serosal fibrosis [8]. We also observed that radiation applied to the tongue led to a reduction of the proliferative marker Ki-67 in the small intestine. Loss of proliferative function affects epithelium development and renders the intestine permeable to luminal bacteria and antigens, potentially exacerbating mucosal inflammation and dysfunction or leading to bacteremia [37]. We also demonstrated that irradiation to the tongue led to reduced ZO-1 in the small intestine. Such damage permits bacterial translocation, potentially contributing to enhancement of the mucositis [38].

On the other hand, severe injury to the mouth mucosa and/or salivary glands after irradiation causes an overall weakening of the animals, which could substantially contribute to the general pathophysiologic manifestations of the body including intestinal toxicity. Such weaken effects might induce small intestine inflammation, an increase of ROS and, consequently, cell death and accumulation of toxic substances within the lumen. Thus, we cannot discard that the gut damage reported in our results might arise secondarily after oral irradiation, promoting epithelial cell breakdown. Other authors have already demonstrated that external radiation of other different organs from the gut, such as the liver, also induces damage in different segments of the rat intestine [7,39]. Therefore, the prevention of severe radiation enteropathy requires protection of the small intestine. Our present results demonstrated significant reductions of these processes in the presence of melatonin. Oral treatment with melatonin gel was associated with improved preservation of small intestinal mucosal architecture. Compare to treatment with vehicle, rats treated with melatonin gel showed significantly greater villus height following tongue irradiation, suggesting that melatonin gel reduced intestinal damage. We cannot exclude an indirect effect of melatonin on this response, by reducing radiation injury in the mouth. However, the gel was topically applied to the rat’s mouth, and the rats swallowed the gel, coating all gastrointestinal mucosa as well as the mouth. Moreover, melatonin is depleted in the mouth and in the intestine after irradiation, and treatment with the gel recovered its physiological levels in both tissues. These findings suggest that the melatonin gel is directly protecting all gastrointestinal mucosa from mucositis.

While the severity of intestinal radiation toxicity is typically determined based on the extent of radiation-induced intestinal crypt cell death, radiation also induces changes in cellular function and activation of the innate immune system, which lead to mucosal breakdown [40]. Our immunohistological studies revealed that the rats treated with melatonin gel also showed increased intestinal Ki-67 and ZO-1 levels, potentially indicating the recovery of intestinal damage as an out-of-field effect of radiation. Moreover, IR in the tongue was associated with oxidative stress in the small intestine, as shown by increased LPO and NO levels, which was counteracted by melatonin gel treatment. Levels of ROS typically increase following chemotherapy or radiation, along with a series of events leading to tissue destruction [41]. Our present results suggest that small intestine injury and apoptosis are also impacted by radiation-induced inhibition of mitochondrial respiratory chain complexes and the ensuing bioenergetics failure induced by mitochondrial dysfunction [42]. IR was associated with decreases of mitochondrial antioxidant enzymes, including GPx, GRd, and SOD, and an increase of GSSG/GSH. Treatment with melatonin gel decreased the mitochondrial oxidative stress, restoring normal levels of mitochondrial complexes.

ROS may promote inflammation by activating multiprotein cytoplasmic complexes inflammasomes, such as NLRP3. Assembly of the NLRP3 inflammasome leads to caspase-1 activation and subsequent cleavage of pro-IL-1β cytokines into their mature forms. Importantly, IL-1β is one of the inflammatory mediators that is reportedly released during mucositis. We recently defined the crucial role of ASC/caspase-1 activation and consequent IL-1β production in the establishment of inflammatory responses in irradiation-induced oral mucositis in rats [24]. This innate immune system pathway activates proinflammatory cytokines produced by NF-κB pathway activation [43]. Our present work also demonstrated the importance of proinflammatory cytokines processed by caspase-1 for the development of irradiation-induced mucositis. We observed a significant increase in the tissue levels of IL-1β, TNF-a, and COX-2. These findings support the hypothesis that NF-κB and NLRP3 work together to activate inflammatory pathways of the innate immune response, resulting in its overstimulation [24]. Since melatonin has antagonist effects on both NF-κB and NLRP3 signaling, it could be a more efficient anti-inflammatory molecule than other drugs. Here we evaluated whether mitochondrial dysfunction participated in NLRP3 inflammasome activation in the small intestine following tongue irradiation. Interestingly, the protective effect of melatonin in mitochondria was associated with inhibition of the NLRP3 inflammasome and decreased mucositis signals.

**Fig 10:**
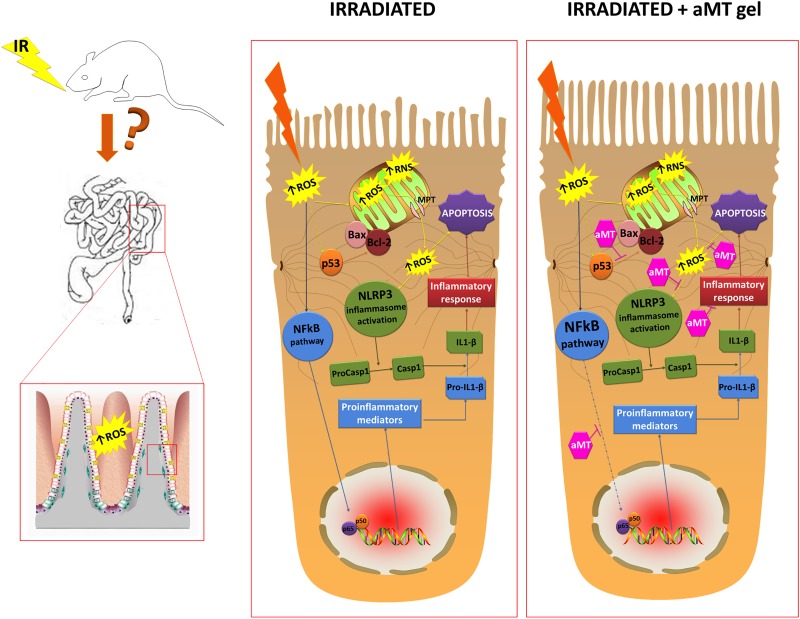
The molecular pathway involved in small intestine damage induced by external radiation, which is inhibited by melatonin gel. Radiation administered to the tongue induced small intestine damage, activating several molecular pathways. Melatonin gel protected mitochondria and inhibited the NF-κB and NLRP3 inflammasome pathways, reducing inflammation and apoptosis, relieving mucosal dysfunction, and facilitating intestinal mucosa recovery.

Studies of apoptotic signaling cascades demonstrate that mitochondria play a major role in regulating the apoptotic process by directly influencing energy production, regulating caspase activation, participating in intracellular calcium ion homeostasis, and releasing ROS [44]. Moreover, exposure to radiation leads to a reduced activity of mitochondrial complexes, accompanied by decreased concentrations of cytochrome c and Bcl-2. Lower levels of the anti-apoptotic Bcl-2 protein facilitate the release of apoptogenic proteins, such as cytochrome c, from the mitochondria into the cytosol, thereby initiating the apoptotic cascade [24]. Correspondingly, here we showed that the radiation-induced inhibition of mitochondrial respiratory chain complexes, and corresponding mitochondrial dysfunction, was accompanied by an increased Bax/Bcl2 ratio. The antiapoptotic benefit of melatonin gel may be attributed to its ability to protect mitochondria in epithelial cells, as well as stem cells, from radiation. The protective effect of melatonin gel in the small intestine was associated with mitochondrial protection and, consequently, with a reduced inflammatory response, decreased intestinal apoptosis, relief of mucosal dysfunction, and facilitated intestinal mucosa recovery (Fig 10).

A clinical trial is currently underway to test melatonin gel for the prevention of oral mucositis in head and neck cancer patients. Interestingly, local application of melatonin gel in the mouth leads to the accumulation of effective concentrations of melatonin in the small intestine to prevent radiotherapy-induced gut toxicity. Additionally, numerous studies show that melatonin has remarkable oncostatic properties, and can reduce tumor promotion and/or progression [45–50]. Thus, the application of melatonin represents an innovative adjuvant strategy in cancer treatment, combining oncostatic and cytoprotective traits. Our present results suggested that treatment with melatonin gel both reduced the toxicity of irradiation and prevented treatment side effects.

## Conclusions

Irradiation of the tongue results in damage to the small intestine, which is outside of the irradiation field. This small intestine damage is likely caused by indirect effects, such as the accumulation of toxic substances within the lumen, promoting epithelial cell breakdown and innate immune system activation [40]. Our present study is unique in that the results indicate that melatonin is a candidate drug that may protect small intestinal cells from the side effects of radiation therapy applied in the oral mucosa.

## Supporting information

S1 File Summary statistics of Fig 1 (Table A). Summary statistics of Fig 2 (Table B). Summary statistics of Fig 3 (Table C). Summary statistics of Fig 4 (Table D). Summary statistics of Fig 6 (Table E). Summary statistics of Fig 7 (Table F) Summary statistics of Fig 8 (Table G). Summary statistics of Fig 9 (Table H).
(DOCX)
