# Supplementary material for: Melatonin protects rats from radiotherapy-induced small intestine toxicity
Source: PLoS One. 2017 Apr 12;12(4):e0174474. doi: 10.1371/journal.pone.0174474 (PMC5389624; doi:10.1371/journal.pone.0174474)
Supplement: S1 File — Summary statistics of Fig 1 (Table A). Summary statistics of Fig 2 (Table B). Summary statistics of Fig 3 (Table C). Summary statistics of Fig 4 (Table D). Summary statistics of Fig 6 (Table E). Summary statistics of Fig 7 (Table F) Summary statistics of Fig 8 (Table G). Summary statistics of Fig 9 (Table H). (DOCX) [file pone.0174474.s001.docx]

**SUPPORTING INFORMATION**

**Table A**

|  | **Control** | | | **IR** | | | **IR +aMT** | | |  |  |  |  | |
| --- | --- | --- | --- | --- | --- | --- | --- | --- | --- | --- | --- | --- | --- | --- |
| **Variables** | **n** | **mean** | **sd** | **n** | **mean** | **sd** | **n** | **mean** | **sd** | **Fexp** | **df** | **P** | **Pairwise** |  |
| **Villuaslenght** | 9 | 889.1 | 151.50 | 11 | 334.5 | 39.25 | 10 | 913.2 | 96.56 | 107.09 | 2;27 | P<0.0001 | a,c | |
| **LPO** | 5 | 0.678 | 0.0734 | 5 | 0.982 | 0.1720 | 5 | 0.816 | 0.0876 | 8.16 | 2;12 | 0.0058 | a | |
| **NO** | 5 | 10.19 | 1.733 | 5 | 19.92 | 3.185 | 4 | 14.75 | 3.140 | 15.86 | 2;11 | 0.0006 | a,c | |

**Table B**

|  | **Control** | | | **IR** | | | **IR +aMT** | | |  |  |  |  |
| --- | --- | --- | --- | --- | --- | --- | --- | --- | --- | --- | --- | --- | --- |
| **Variables** | **n** | **mean** | **sd** | **n** | **mean** | **sd** | **n** | **mean** | **sd** | **Fexp** | **df** | **P** | **Pairwise** |
| **KI** | 10 | 34.2 | 4.00 | 10 | 27.3 | 3.82 | 10 | 34.7 | 3.97 | 11.06 | 2;27 | 0.0003 | a,c |
| **ZO** | 5 | 2012919.0 | 449962.90 | 6 | 447588.8 | 321096.20 | 6 | 1916448.0 | 750394.80 | 15.06 | 2;14 | 0.0003 | a,c |

**Table C**

|  | **Control** | | | **IR** | | | **IR +aMT** | | |  |  |  |  |
| --- | --- | --- | --- | --- | --- | --- | --- | --- | --- | --- | --- | --- | --- |
| **Variables** | **n** | **mean** | **sd** | **n** | **mean** | **sd** | **n** | **mean** | **sd** | **Fexp** | **df** | **P** | **Pairwise** |
| **CI** | 4 | 1.10 | 0.207 | 3 | 0.30 | 0.116 | 3 | 1.32 | 0.250 | 107.090 | 2;27 | P<0.0001 | a,c |
| **CII** | 4 | 1.31 | 0.358 | 3 | 0.33 | 0.262 | 4 | 1.53 | 0.275 | 14.295 | 2;8 | 0.0023 | a,c |
| **CIII** | 4 | 1.18 | 0.215 | 4 | 0.42 | 0.199 | 4 | 0.85 | 0.295 | 10.106 | 2;9 | 0.0050 | a |
| **CIV** | 4 | 1.21 | 0.400 | 4 | 1.19 | 0.316 | 4 | 1.21 | 0.401 | 0.002 | 2;9 | 0.9977 |  |
| **CV** | 4 | 1.08 | 0.091 | 4 | 0.80 | 0.061 | 4 | 1.29 | 0.106 | 31.038 | 2;9 | 0.0001 | a,b,c |

**Table D**

|  | **Control** | | | **IR** | | | **IR +aMT** | | |  |  |  |  |
| --- | --- | --- | --- | --- | --- | --- | --- | --- | --- | --- | --- | --- | --- |
| **Variables** | **n** | **mean** | **sd** | **n** | **mean** | **sd** | **n** | **mean** | **sd** | **Fexp** | **df** | **P** | **Pairwise** |
| GPx | 7 | 133.59 | 25.765 | 7 | 88.14 | 19.944 | 6 | 131.97 | 18.639 | 9.54 | 2;27 | 0.0017 | a,c |
| GPx_WB | 6 | 1.00 | 0.199 | 6 | 0.70 | 0.147 | 4 | 1.45 | 0.459 | 9.14 | 2;13 | 0.0033 | b,c |
| **GRd** | 4 | 9.81 | 1.138 | 7 | 6.49 | 1.235 | 7 | 10.55 | 1.782 | 15.32 | 2;16 | 0.0002 | a,c |
| GRd_WB | 3 | 1.08 | 0.219 | 4 | 0.62 | 0.173 | 4 | 1.55 | 0.247 | 19.06 | 2;8 | 0.0009 | a,b,c |
| GSH | 6 | 1.31 | 0.237 | 7 | 0.52 | 0.187 | 4 | 1.07 | 0.069 | 29.52 | 2;14 | 0.0001 | a,c |
| GSH_GSSG | 7 | 0.53 | 0.241 | 4 | 2.63 | 0.857 | 4 | 0.59 | 0.110 | 29.25 | 2;12 | 0.0000 | a,c |
| GSSG | 6 | 0.52 | 0.218 | 6 | 0.91 | 0.216 | 5 | 0.58 | 0.095 | 7.20 | 2;14 | 0.0071 | a,c |
| SOD | 3 | 10.59 | 0.960 | 4 | 5.71 | 0.569 | 4 | 10.28 | 0.795 | 47.96 | 2;8 | 0.0000 | a,c |
| SOD_WB | 6 | 0.90 | 0.531 | 6 | 1.37 | 0.319 | 4 | 3.41 | 1.152 | 17.90 | 2;13 | 0.0002 | b,c |

**Table E**

|  | **Control** | | | **IR** | | | **IR +aMT** | | |  |  |  |  |
| --- | --- | --- | --- | --- | --- | --- | --- | --- | --- | --- | --- | --- | --- |
| **Variables** | **n** | **mean** | **sd** | **n** | **mean** | **sd** | **n** | **mean** | **sd** | **Fexp** | **df** | **P** | **Pairwise** |
| **Caspase_1** | 4 | 1.10 | 0.083 | 3 | 4.72 | 0.152 | 4 | 0.70 | 0.125 | 1131.59 | 2;8 | P<0.0001 | a,b,c |
| **NLRP3** | 6 | 1.06 | 0.120 | 6 | 1.89 | 0.106 | 6 | 1.13 | 0.086 | 114.84 | 2;15 | P<0.0001 | a,c |
| **Procaspase_1** | 5 | 1.16 | 0.053 | 5 | 0.25 | 0.106 | 5 | 0.61 | 0.123 | 108.04 | 2;12 | P<0.0001 | a,b,c |

**Table F**

|  | **Control** | | | **IR** | | | **IR +aMT** | | |  |  |  |  |
| --- | --- | --- | --- | --- | --- | --- | --- | --- | --- | --- | --- | --- | --- |
| **Variables** | **n** | **mean** | **sd** | **n** | **mean** | **sd** | **n** | **mean** | **sd** | **Fexp** | **df** | **P** | **Pairwise** |
| **IL_1beta** | 4 | 0.91 | 0.250 | 3 | 1.52 | 0.277 | 4 | 1.08 | 0.071 | 5.69 | 2;11 | 0.0201 | c |
| **NF_KB_nuc** | 6 | 0.85 | 0.306 | 6 | 6.15 | 0.234 | 3 | 1.38 | 0.044 | 8.16 | 2;12 | 0.0058 | a,b,c |
| **NF_kB_cit** | 6 | 1.12 | 0.326 | 5 | 12.71 | 1.211 | 3 | 8.16 | 0.311 | 15.86 | 2;11 | 0.0006 | a,b,c |
| **TNF_alfa** | 4 | 0.91 | 0.250 | 3 | 1.52 | 0.277 | 4 | 1.08 | 0.071 | 7.52 | 2;8 | 0.0145 | a |

**Table G**

|  | **Control** | | | **IR** | | | **IR +aMT** | | |  |  |  |  |
| --- | --- | --- | --- | --- | --- | --- | --- | --- | --- | --- | --- | --- | --- |
| **Variables** | **n** | **mean** | **sd** | **n** | **mean** | **sd** | **n** | **mean** | **sd** | **Fexp** | **df** | **P** | **Pairwise** |
| Bax | 6 | 0.99 | 0.118 | 6 | 6.54 | 0.388 | 6 | 3.17 | 0.313 | 536.54 | 2;11 | 0.0000 | a,b,c |
| Bax_Bcl2 | 4 | 0.96 | 0.036 | 4 | 25.41 | 5.072 | 5 | 0.63 | 0.039 | 108.91 | 2;10 | 0.0000 | a,c |
| Bcl2 | 5 | 0.99 | 0.069 | 5 | 0.27 | 0.047 | 5 | 0.63 | 0.039 | 230.79 | 2;12 | 0.0000 | a,b,c |
| P53 | 5 | 1.01 | 0.065 | 4 | 3.02 | 0.619 | 5 | 1.26 | 0.341 | 34.74 | 2;11 | 0.0000 | a,c |

**Table H**

|  | **Control** | | | **IR** | | | **IR +aMT** | | |  |  |  |  |
| --- | --- | --- | --- | --- | --- | --- | --- | --- | --- | --- | --- | --- | --- |
| **Variables** | **n** | **mean** | **sd** | **n** | **mean** | **sd** | **n** | **mean** | **sd** | **Fexp** | **df** | **P** | **Pairwise** |
| Mel | 4 | 4.8 | 0.88 | 3 | 5.8 | 1.47 | 4 | 15.6 | 2.02 | 59.0174 | 2;8 | P<0.0001 | b,c |
